# Coverage and quality of patient participation groups: a mixed-methods analysis of Care Quality Commission reports

**DOI:** 10.3399/BJGP.2025.0088

**Published:** 2026-03-10

**Authors:** Emily Jane Boam, Luke Munford, Claire Planner, Bruno Rushforth, Jessica Drinkwater

**Affiliations:** 1 Health Research and Statistics, Centre for Primary Care and Health Services Research, University of Manchester, Manchester, UK; 2 Public Health Registrar, NHS England Yorkshire and Humber School of Public Health, Yorkshire, UK; 3 Centre for Primary Care and Health Services Research, University of Manchester, Manchester, UK; 4 Foundry Lane Surgery, Seacroft, Leeds, UK; 5 Future Leaders programme supervisor and locality lead, for GP Recruitment and Careers, NHS England Yorkshire and Humber GP School, UK

**Keywords:** Care Quality Commission, deprivation, general practice, health inequalities, health inequities, patient participation groups, primary health care

## Abstract

**Background:**

Patient participation in health systems is increasing globally. English general practices have been required to establish patient participation groups (PPGs) since 2015. However, little is known about current PPG coverage and distribution.

**Aim:**

To explore the relationship between PPG coverage and quality with general practice deprivation deciles, geographical location, and Care Quality Commission (CQC) ratings.

**Design and setting:**

Mixed-methods documentary analysis of quantitative and qualitative data was carried out from general practice CQC reports in Yorkshire and Humber, England.

**Method:**

Data were extracted from CQC reports for practices in the most and least deprived areas across three integrated care systems (ICSs). Quantitative data examined PPG coverage by practice deprivation decile, location, and CQC rating. Qualitative thematic analysis assessed PPG quality.

**Results:**

Of 122 practices, 99 (81.1%) had a PPG, 16 (13.1%) lacked one, and seven (5.7%) had a partial PPG. Practices in the most deprived areas were significantly less likely to have a PPG than those in the least deprived areas (*P* = 0.006). While there were minor differences in PPG coverage between ICS locations, no significant variation was observed based on CQC ratings. PPG activities included workforce adjustments and training, infrastructure, communications, accessibility and appointments, health promotion and education, and fundraising.

**Conclusion:**

Practices in the most deprived areas were significantly less likely to have a PPG (*P* = 0.006), and their PPGs engaged in different activities compared with those in the least deprived areas. This may exacerbate inequalities as PPGs are one mechanism to support patient participation and improve general practice quality and experience.

## How this fits in

One stated aim of patient participation groups (PPGs) is to improve general practice quality, but there is limited data on their coverage or effectiveness. This study reveals a significant disparity in PPG coverage in more deprived compared with less deprived areas (*P* = 0.006). It also highlights differences in PPG activities, which may reflect resource availability. Policymakers should be aware of these findings and prioritise equitable and tailored strategies to support and sustain PPGs in socioeconomically deprived areas, to enhance patient participation and reduce inequalities in healthcare quality.

## Introduction

Globally, patient participation is increasingly recognised as an integral part of high-quality health care. This is reflected in global policy frameworks and healthcare reforms aimed at achieving more equitable, person-centred care. However, the emphasis placed on patient involvement and its implementation varies considerably.^1–3^ The World Health Organization (WHO) urges member states to strengthen, systemise, and sustain patient participation within healthcare system planning and resource allocation^1^. In England, the NHS Constitution emphasises the importance of patient participation in healthcare policy and service delivery.^4^ One mechanism for achieving this in general practice is having a patient participation group (PPG). Since 2015, the English GP contract has mandated that all practices establish and maintain a PPG representing the practice population, and engage with it regularly throughout the year to gather feedback on services.^5–7^

PPGs can have an important role in the quality of general practice, contributing through a variety of potential functions. First, they provide a platform for patient perspectives to be considered in decision making about services they receive, resulting in more appropriate services.^8,9^ Second, they act as a route for constructive dialogue and feedback, highlighting areas for development, contributing to action plans and monitoring improvements.^8–10^ Third, PPGs may encourage community engagement and collaboration, for example, by introducing peer-led services to meet local needs.^9,11^ Finally, they may disseminate health awareness, education, and empowerment messages, ultimately improving health outcomes and satisfaction.^8–10^ Overall, PPGs have the potential to facilitate patient-centred care, collaboration, and continuous improvement in general practice services.

Despite this potential to improve quality, there are little data on the coverage, functions, or effectiveness of PPGs in practice. Coverage was most recently explored in a 2016 study, which estimated that only one-quarter of general practices had a PPG.^9^ Evidence suggests that PPGs struggle to recruit and maintain membership, particularly within urban areas.^12–16^

Barriers to establishing a PPG are varied and are thought to include lack of practice time and resources, perceived lack of interest from patients, difficulties working with a diverse patient population, and fear that the group will be unrepresentative.^10,14,17^ These last two barriers raise concerns about inequalities in participation and outcomes related to PPGs. In particular, it is known that participating in community assets (of which PPGs are one) improves quality of life and reduces healthcare costs, and there are fewer community assets in areas of high socioeconomic deprivation.^18,19^

This article aims to examine whether the presence and quality of PPGs vary by deprivation decile and geographical area, and whether the Care Quality Commission (CQC) ratings are associated with PPG presence.

## Method

### Study design

This study used a mixed-methods descriptive design, incorporating quantitative and qualitative analysis using freely available, routinely collected CQC report data.

### Data collection

The CQC is the independent regulator of health and social care in England.^20^ It monitors quality in general practice, including *‘involving and engaging with the patient population and communities’* within the ‘well-led’ domain of its assessment framework.^21–23^ The CQC publishes publicly available reports on the general practices it inspects and is the only organisation to publish data regarding PPGs.

The Data and Analytics department of NHS England provided a list of all general practices within the North East and Yorkshire region. This was used to generate a list of all practices in the three integrated care system (ICS) areas of Yorkshire and Humber: Humber and North Yorkshire, West Yorkshire, and South Yorkshire. Yorkshire and Humber was chosen as it represents a distinct yet sizeable region, ideal for comparison between ICSs. The area encompasses approximately 5.5 million people and around 604 general practices.^24,25^ Its manageable size facilitates data collection while offering diverse demographics, including urban centres — Leeds, Sheffield, Bradford, Hull, and York — and rural communities, such as those in the Yorkshire Dales and North York Moors. This diversity enables meaningful comparisons across areas of varying deciles of deprivation.

Decile of deprivation was measured by the Index of Multiple Deprivation (IMD) from the English Indices of Deprivation 2019 as the official measure of deprivation for small areas in England.^24^ Each area in England is ranked from most deprived to least deprived, and the data are split into deciles, for example, the most deprived 10% of areas into decile 1. General practices located in areas corresponding to IMD decile 1 (most deprived) and 10 (least deprived) were included to enable a direct comparison between the extremes of deprivation while maintaining a manageable dataset size.

Between August 2023 and February 2024, data were collected and analysed by one research team member (the first author). The CQC website search function was used to identify each practice’s most recent CQC full inspection report. Practices were excluded if there were no CQC data, for example, because a new provider had taken over the practice, data were archived because the practice had ceased to exist, or the most recent full inspection report was dated before the PPG contract change on 1 April 2015.

The following quantitative data were extracted from each report and managed in Microsoft Excel: date of report publication; presence of a PPG (categorised as yes, no, or partial by the CQC); overall practice CQC rating; and CQC well-led domain rating.

Each inspection report was hand-searched for qualitative data, as an indicator of PPG quality, using the following keywords: participation, engagement, involvement, reference, group, PPG, PRG (patient reference group), and community. The reports were then reviewed for additional details related to patient participation.

### Analysis

Quantitative data analysis was performed in Stata (version 17). First, the data were analysed using Fisher’s exact test to compare PPG’s presence, partial presence, or absence with IMD decile, geographical area, and CQC rating individually. Then, multivariable regression analysis was used to compare PPG presence (yes or partial) versus absence, with IMD decile and geographical area together.

Qualitative data were extracted from the reports and managed in Microsoft Excel. Data were categorised into two major themes regarding the format of the PPG (type and regularity of meetings) and PPG activities. Thematic analysis was then used to further categorise the PPG activities into sub-themes.

## Results

### Quantitative data

#### Practice characteristics

One-hundred and forty-six practices were identified across the three ICSs conditional on being in either decile 1 IMD or decile 10 IMD areas. Twenty-two practices in decile 1 IMD areas were removed: 15 practices had recently changed provider and had not yet been inspected, five practices had recently been set up and had not yet received an inspection, and two practices had changed addresses since their last inspection. Two practices in decile 10 IMD areas were removed: one had a most recent CQC full inspection report before 1 April 2015, and one had recently changed providers and had not yet been inspected.

After removing these practices, the sample totalled 122 practices, including 86 in decile 1 IMD areas and 36 in decile 10 IMD areas. The fact that there were many more practices in the most deprived deciles reflects the fact that, on average, Yorkshire and the Humber is one of the more deprived parts of England.

#### CQC report characteristics

Report publication dates ranged from 29 July 2015 to 16 September 2023. Owing to the large time period analysed, the style and length of CQC reports varied from a single PDF document containing all information in written prose to two separate PDF documents, including a full inspection report and evidence table. Although the style of reports varied, the content and phrases used remained the same, for example, *‘has an active PPG’*.

#### PPG status

Overall, 99 practices (81.1%) had a PPG, 16 practices (13.1%) did not have a PPG, and seven (5.7%) had a partial PPG. The CQC did not define ‘partial’ in the reports, but this referred to PPGs shared across practices within the same primary care network (PCN) and PPGs that were paused during the COVID-19 pandemic and had yet to restart.

#### The association between PPG status and deprivation decile

A significantly smaller proportion of practices in decile 1 IMD areas had a PPG (75.6%, *n* = 65) compared with practices in decile 10 IMD areas (94.4%, *n* = 34) (*P* = 0.038; Table 1).

**Table 1.** Association between patient participation group (PPG) status and deprivation decile

Integrated care system	Yes		No		Partial		Total
	Decile 1	Decile 10	Decile 1	Decile 10	Decile 1	Decile 10	
Humber and North Yorkshire	9	16	5	1	0	0	31
West Yorkshire	36	11	5	0	3	0	55
South Yorkshire	20	7	5	0	3	1	36
Total	65	34	15	1	6	1	122

The conditional difference, accounting for ICS location and IMD decile, was more significant (*P* = 0.006), with practices in the most deprived areas 19.4 percentage points less likely to have a PPG than practices in the least deprived areas (Table 2).

**Table 2.** Multivariable regression: association between patient participation group status and deprivation decile and integrated care system location

	Effect size	*P*-value	95% confidence interval
PPG (decile 1 IMD versus decile 10 IMD [reference group])	–0.19	0.006	–0.33 to 0.06
ICS (South Yorkshire versus Humber and North Yorkshire [reference group])	0.12	0.163	–0.05 to 0.28
ICS (West Yorkshire versus Humber and North Yorkshire [reference group])	0.17	0.031	0.02 to 0.32
_cons	0.89	<0.001	0.76 to 1.00

ICS = integrated care system. IMD = Index of Multiple Deprivation. PPG = patient participation group.

#### The association between PPG status and ICS location

Practices in West Yorkshire were most likely to have a PPG (85.5%, *n* = 47), followed by Humber and North Yorkshire (80.6%, *n* = 25), and then South Yorkshire (75.0%, *n* = 27). However, the conditional difference, simultaneously accounting for IMD decile and ICS location, found practices in West Yorkshire were 17.0 percentage points more likely to have a PPG than practices in Humber and North Yorkshire (*P* = 0.030; Table 2).

#### CQC rating

CQC overall rating varied from ‘outstanding’ to ‘inadequate’. All practices had the same rating for the well-led domain as their overall rating, except one practice in a decile 1 IMD area, which was rated good overall and outstanding for the well-led domain. This practice had a PPG.

#### The association between PPG status and CQC rating

Of the 16 practices without PPGs, 12 practices were rated good (75.0%), two were rated as requiring improvement (12.5%), and two as inadequate (12.5%). Of the seven practices with a partial PPG, all were rated good. Overall and well-led ratings were the same for all practices without a PPG and with a partial PPG (Table 3).

**Table 3.** Association between patient participation group status and overall Care Quality Commission (CQC) rating

Patient participation group status	Outstanding		Good		Requires improvement		Inadequate		Total
	Decile 1	Decile 10	Decile 1	Decile 10	Decile 1	Decile 10	Decile 1	Decile 10	
Yes	1	1	60	33	4	0	0	0	99
No	0	0	11	1	2	0	2	0	16
Partial	0	0	6	1	0	0	0	0	7

There was no significant difference in overall or well-led CQC ratings between practices with a PPG, without a PPG, and with a partial PPG (*P* = 0.053 and *P* = 0.054).

### Qualitative data

#### How did the CQC reports illustrate the quality of PPGs? PPG format

A minority of reports described how often PPGs met, the number of members, and whether they met online or face to face.

Thirty-four practices were described as having regular PPG meetings. Only eight reports quantified the regularity of meetings, ranging from monthly to 6-monthly. Five reports indicated the number of PPG members, ranging from eight members of a face-to-face PPG to 300 members of a virtual PPG. Nine reports described entirely virtual PPGs, and two described entirely face-to-face PPGs. Three reports described hybrid PPGs, two with regular face-to-face meetings and virtual meetings as required, and one (the practice above) with 300 virtual PPG members and a core committee of eight who met face to face more regularly.

#### PPG activities

PPGs engaged in workforce adjustments and training, infrastructure, communications, accessibility and appointments, health promotion and education, and fundraising. Differences in PPG activities between decile 1 and decile 10 IMD areas were most pronounced within workforce adjustments and training, health promotion and education, and fundraising (Supplementary Table S1).

a) Workforce adjustments and training: PPG feedback informed workforce adjustments and training in nine practices within decile 1 IMD areas. PPGs were involved in the recruitment process for a salaried GP, nurse, pharmacist, and Bengali-speaking staff member. Staff training courses recommended by PPGs included customer care, conflict resolution, and dementia awareness. Other examples where PPG feedback influenced workforce adjustments included diverting more staff from other roles to answer the telephone in the morning.

The only example of workforce adjustment changes within decile 10 IMD areas was the introduction of staff name badges in one practice.

b) Infrastructure: There were 33 examples, spread evenly across decile 1 and decile 10 IMD areas, where PPG feedback led to changes in practice infrastructure. Changes primarily involved updating noticeboards, TV screens, and signage in reception and waiting areas. Improvements for patient confidentiality included relocating consultation rooms, installing sound-proof curtains, and adding a radio to mask conversations. Specific changes for particular patient groups included creating children’s waiting areas and a separate room for vulnerable patients.
c) Communications: Twenty CQC reports described PPG-led changes to practice communication. Examples included developing or improving telephone systems, practice websites, newsletters, social media pages, letters, and text messages. These changes were consistently observed across decile 1 and decile 10 IMD areas.
d) Accessibility and appointments: There were 15 examples where PPG feedback resulted in increased access to appointments. This mainly focused on increasing the number of urgent appointments but also included routine appointments, appointments with female GPs, and extended hours. Within decile 10 IMD practices, several changes were made to increase accessibility for particular patient populations, including longer appointments for patients with learning disabilities and increased home visits for older patients. Two practices described adopting a new process of GP triage.
e) Health promotion and education: Thirty examples of PPG involvement in health promotion and education activities were given. These included promoting, coordinating, and stewarding annual vaccination days and health promotion events. In four practices within decile 1 IMD areas, the PPG suggested practical support for vulnerable patients, provided by practice staff volunteering at weekends. This support included a soup kitchen, food parcels, and winter packs. Three practices in decile 10 IMD areas described promoting physical activity through a practice gym and patient-led running groups. Two practices in decile 10 IMD areas provided PPG-led basic life-support courses.
f) Fundraising: Seven reports described PPG fundraising. Only one practice in a decile 1 IMD area reported fundraising, by setting up a bookstall for charity. All other examples were in decile 10 IMD areas and included fundraising for medical equipment, including two defibrillators, and children’s toys for the waiting room.

### Practices without PPGs

Two reports of practices without PPGs described barriers to recruiting and maintaining a diverse PPG membership.

*‘As with many of the practices within the PCN, the practice had struggled to develop and maintain an active patient participation group. We were informed of the challenges the practice had due to the patient demographics, which included language barriers, illiteracy, deprivation, poverty and multi-occupancy of premises within the area*.’ (Practice 8, decile 1 IMD area, West Yorkshire ICS)

Four reports of practices without PPGs described ways that practices attempted to recruit members, including working with other local practices and advertising via an open day, posters in the waiting room, and on their websites.

## Discussion

### Summary

General practices in the most socioeconomically deprived areas are significantly less likely to have a PPG than those in the least deprived areas (*P* = 0.006). PPG coverage showed minor variations between ICS locations, potentially owing to current and historical variations in local support and policies. This suggests that socioeconomic deprivation, rather than location, is a key factor in determining whether practices have a PPG.

There was no difference in CQC ‘overall’ and ‘well-led’ ratings between practices with or without a PPG, despite PPGs being a contractual requirement. This suggests that having a PPG does not influence CQC ratings. The qualitative data reveal variation in PPG format and activities, which could affect the quality of care. These activities were grouped into the following six broad categories: workforce adjustments and training; infrastructure; communications; accessibility and appointments; health promotion and education; and fundraising. Differences in activities were linked to decile of deprivation. PPGs in less deprived areas focused on fundraising for extra equipment and promoting physical activity, while those in more deprived areas focused on initiatives such as soup kitchens and winter packs. Although these activities may target local needs, the disparity in PPG coverage and activities could widen inequalities in healthcare quality.

### Strengths and limitations

Access to information regarding PPGs is challenging to obtain as it is difficult to contact PPGs independently. The study used freely accessible information to include a comprehensive sample of practices in Yorkshire and Humber and all decile 1 and decile 10 IMD general practices across all ICS areas. However, being limited to one region, we must be cautious about extrapolating these trends nationally.

Some methodological limitations should be noted. Although IMD decile does allow comparison between areas, it does not provide a quantitative scale, for example, a decile 1 area is not necessarily 10 times more deprived than a decile 10 area. Furthermore, IMD decile is designed to identify aspects of deprivation, not affluence; a decile 10 area may be the least deprived in the country, but it is not necessarily among the most affluent.^26,27^ In addition, we used the IMD rank (and hence decile) of the postcode of the general practice. We could not account for the overall deprivation composition of the general practice’s list. It is possible that, while the practice is in a deprived area, patients from less deprived areas attend and vice versa. However, the fact we only use the two extreme deciles means this is much less likely to be a problem as it is uncommon (outside of London) to have areas of extremely high deprivation clustered near areas of extremely low deprivation.

Additionally, the CQC reports lacked standardisation concerning PPG details. No formal definition was given to define ‘yes’, ‘no’, or ‘partial’ concerning PPG coverage and descriptions of PPG activities varied considerably between reports. CQC reports also rely on subjective accounts from patients and practice staff, who are aware that the CQC inspection is a quality judgement. This awareness likely introduces reporting bias, as individuals may emphasise successes. Nonetheless, we have compared across CQC reports rather than between entirely different datasets and still identified notable differences.

Another limitation is the CQC’s role as a quality assessor rather than an enforcer of general practice contracts. The CQC ratings framework does not require practices to demonstrate every rating characteristic. For example, a practice lacking a PPG might still receive a ‘good’ rating if its absence does not significantly impact care quality or patient experience.^28^ This may explain why PPG status did not appear to influence CQC ratings.

Lastly, PPGs are just one of the mechanisms the CQC uses to evaluate whether general practices actively engage with patients’ views. The CQC’s single-assessment framework emphasises broader responsibilities within the ‘partnerships and communities’ aspect of the well-led domain. This calls for collaboration with individuals, communities, stakeholders, and organisations to share knowledge, foster innovation, and deliver integrated care that improves patient outcomes.^29,30^ Therefore, it is possible that practices without a formal PPG are engaging with patients through alternative methods, with the CQC inspection emphasising overall patient involvement, which could encompass other forms of feedback. However, the reports analysed in this study did not provide evidence to support this assertion.

### Comparison with existing literature

There has been no data exploring the coverage of PPGs since 2016. This may be because it is assumed that all practices have a PPG since it became mandatory in the 2015 general practice contract. Therefore, our finding that 13.1% of general practices did not have a PPG and that these are more likely to be in socioeconomically deprived areas is important. Previous research suggests urban areas are less likely to have a PPG.^14^ This paper only explored coverage by urban and rural location, and did not report on deprivation deciles. However, government data show that 12% of people living in urban areas are within IMD 1 compared with 1% of rural areas.^27^ Therefore, it is possible that the 2016 findings were related to deprivation, which is consistent with our findings. While our study suggests the proportion of practices with a PPG has increased markedly since the 2016 study, it remains substantially below what might reasonably be expected given contractual requirements.

A minority of CQC reports from practices in socioeconomically deprived areas without a PPG highlighted barriers to establishing and maintaining a PPG, including language barriers, illiteracy, deprivation, poverty, and multi-occupancy of premises. This is consistent with previous research, which reported a perceived lack of patient interest and difficulties working with a diverse patient population.^10,14,15^ These barriers are more prevalent in more deprived areas compared with affluent areas.

To avoid widening inequality in participation, other authors have argued for moving away from PPGs to different modes of patient engagement with structurally disempowered groups (those disadvantaged by systems, laws, institutions, and policies) that recognise and address power differentials, mistrust, and previous trauma, including trauma inflicted by healthcare services.^31–34^ These different modes of engagement often require skilled facilitation, participatory mechanisms, and long-term investment of time to overcome mistrust and build equitable relationships.^17,31–33^ Therefore, future work should address structural barriers to participation by disempowered groups by moving beyond outreach and towards equity-focused practices that challenge existing power dynamics embedded in many current models, including some PPGs.

Patients are now widely recognised as important partners in the design and delivery of health care.^3^ Our study highlighted several key activities through which PPGs can help improve the quality of general practice, including contributions to workforce adjustments and training, infrastructure, communication, accessibility and appointment management, health promotion and education, and fundraising.

Previous research has reported the outcomes of patient participation in two key areas: as collaborators within discrete projects, such as developing education tools for patients and providers, and informing policy documents;^35–37^ and within larger structural processes, such as informing service delivery and governance.^21,38,39^

Other research has categorised the positive impact of patient participation on healthcare organisations into the following three themes: impact at the decile of quality of care (and research); impact at the decile of the team and organisation; and impact at the decile of the individuals participating.^40^ Most of the descriptions of PPG impact within the CQC reports focused on the impact at the decile of quality of care and the impact at the decile of the team and organisation. There was no information in the reports about the impact of the PPG at the decile of the individuals participating. Little is known about who PPG members are, why they join PPGs, and their experiences of being involved.

### Implications for practice

The qualitative data in the CQC reports suggest that PPGs have a decision-making role in general practice and influence service delivery, potentially enhancing quality of care. However, the presence of a PPG did not affect CQC ratings, suggesting that, despite being a contractual requirement and reported on by the CQC, it does not contribute to their assessment of quality. PPG members that the authors (the first and fifth authors) work with feel let down by the lack of value being placed on PPGs as an indicator of quality and the lack of oversight of GP contractual requirements in this regard. Therefore, we recommend the standardisation of CQC reports with respect to PPG and patient engagement criteria, and the revision of inspection criteria to include and acknowledge alternative engagement methods if the CQC is indeed utilising these to form part of its quality assessment.

Although enforcing contractual requirements is not the responsibility of the CQC, it remains unclear which NHS body holds this accountability. It may be that integrated care boards (ICBs) hold — or should hold — this responsibility, but, in practice, this commonly amounts to no more than general practices ticking a box to self-declare they have a PPG, with little clarity on the consequences of not doing so. While the CQC appears to assess the quality and effectiveness of PPGs, it does not appear to act on these assessments. This lack of clarity risks undermining trust in both general practice accountability and CQC processes.^41–44^ Compounding these concerns, a recent independent review has identified serious internal issues within the CQC itself, including insufficient clinical expertise among inspectors, inconsistencies in assessments, and technical challenges within the CQC’s IT system.^44^ Together, these issues may erode confidence in regulatory oversight and raise questions about the effectiveness of healthcare accountability.

Marmot describes one of the principles needed to address health inequalities as creating and developing healthy and sustainable communities.^45^ These are communities where people participate collectively, building social capital, so policies are shaped by the experiences of those most affected by them.^46^

Deprived neighbourhoods without active and engaged communities, often referred to as ‘left behind neighbourhoods’, experience worse health outcomes than similarly deprived areas with strong community networks.^47^ Therefore, strong communities can mitigate some effects of deprivation, although it takes considerable time and resources for communities to organise and make a measurable impact. This has significant implications for general practice and PPGs in left behind neighbourhoods, where supporting and maintaining a PPG will require more time and resources, yet potentially have more impact.

Time and resources to strengthen PPGs are essential to avoid tokensim by moving from passive consultation towards partnership and co-production, to challenge structural barriers, and to meaningfully involve those whose voices are often absent from influencing decision making.^33^ As community assets embedded within communities and employing local staff, general practices are well-positioned to encourage engagement by promoting active participation through initiatives such as PPGs. However, achieving this requires a shift in health policy around PPGs, from a universal approach (all practices should have PPGs) to a proportionate universal approach where funding is increased for practices in areas of high socioeconomic deprivation and especially left behind neighbourhoods in recognition of the additional time and resources required.^47^ This will help ensure practices better meet the needs of the populations they serve, which is essential to addressing health service and health inequalities, and it may also support broader community development work. 
